# Effect of citronellol on oxidative stress, neuroinflammation and autophagy pathways in an *in vivo* model of Parkinson's disease

**DOI:** 10.1016/j.heliyon.2022.e11434

**Published:** 2022-11-03

**Authors:** Richard L. Jayaraj, Sheikh Azimullah, Khatija A. Parekh, Shreesh K. Ojha, Rami Beiram

**Affiliations:** Department of Pharmacology and Therapeutics, College of Medicine and Health Sciences, United Arab Emirates University, Al Ain, United Arab Emirates

**Keywords:** Neuroprotection, Dopaminergic neurons, Citronellol, Rotenone, Apoptosis

## Abstract

Citronellol, a monoterpene found in the essential oils of Cymbopogo plants has been reported to possess various biological properties. In the present study, we investigated the neuroprotective mechanisms of citronellol against rotenone induced neurodegeneration by using rat model of Parkinson's disease (PD). Our results demonstrated that oral administration of citronellol prevented rotenone induced reactive oxygen species production, lipid peroxidation and enhanced Nrf2 expression, catalase, glutathione peroxidase and superoxide dismutase levels in the brain. Enzyme-linked immunosorbent assays showed that citronellol reduced secretion of TNF-α, IL-1β, IL-6 and decreased MMP-9 expression levels. Further, citronellol prevented rotenone induced microglia (Iba-1 staining) and astrocyte (GFAP staining) activation. Western blot analysis showed that citronellol significantly decreased the expression of cyclooxygenase-2 and inducible nitric oxide synthase-2 that are key markers of neuroinflammation. We further evaluated the effect of citronellol on dopaminergic neurons in substantia nigra pars compacta (SNpc) and striatum (ST) which are key anatomical structures in PD. Tyrosine hydroxylase (TH) immunoreactivity showed that citronellol preserved Tyrosine hydroxylase (TH) positive dopaminergic neurons and enhanced TH striatal expression levels significantly compared to rotenone alone group. Further, to understand the effect of citronellol on apoptosis and proteotoxicity, we evaluated apoptotic markers (Bax, Bcl-2), growth regulator (mTOR) and α-synuclein expression. Citronellol attenuated rotenone induced expression of pro-apoptotic protein Bax, reduced α-synuclein expression and enhanced Bcl-2 and mTOR levels. In addition, citronellol modulated autophagy pathway by decreasing LC-3 (Microtubule-associated proteins) and p62 levels. Taken together, our results demonstrate that citronellol protected dopaminergic neurons through its antioxidant, anti-inflammatory, anti-apoptotic and autophagy modulating properties.

## Graphical abstract

**Figure:**
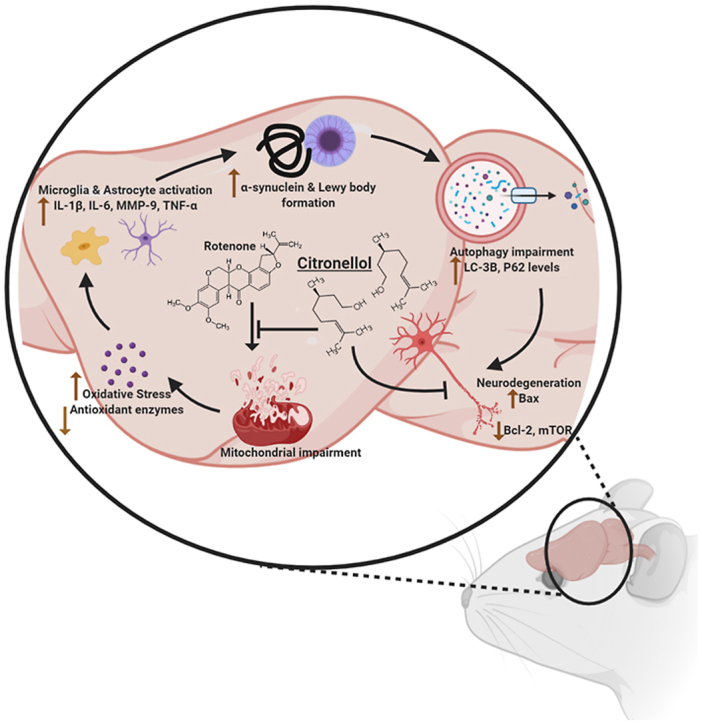

## 1 Introduction

Parkinson's disease, the second most common age-related neurodegenerative disease following Alzheimer's, affects 1% of the human population aged over 60 and its incidence increases with age [1]. Clinically, PD is characterized by presence of motor impairments such as bradykinesia, rigidity, tremor and postural abnormalities. Nevertheless, few non-motor symptoms including olfactory, cognitive and emotional anomalies are early features in PD [2]. Recent study reported that the total economic burden (direct and indirect medical cost) of PD in the United States accounts for US$51.9 billion per million PD patients and it is projected to increase to US$79 billion by 2037 [3]. Global population ageing report states that the ageing population of United Arab Emirates (UAE) has been projected to increase from 5.1% in 2000 to 26.7% by 2050. This alarming increase in UAE and globally will reciprocally increase the incidence of neurological disorders such as PD [4]. However, due to the inability to find curative therapies, global epidemiologic predictions are not hopeful [5]. Pathologically, PD occurs due to dopaminergic neuronal loss in substantia nigra pars compacta with resultant loss of dopamine in striatal terminals. Oxidative stress and neuroinflammation are critical players in idiopathic PD development and progression. Dopaminergic neurons are particularly prone to oxidative injury due to their ability to produce reactive oxygen species during dopamine metabolism or during auto-oxidation of dopamine. Therefore, production of antioxidants that are transcriptionally regulated by NF-E2 related factor-2 (Nrf2) plays a major role in neuroprotection [1]. Disturbance in anti-oxidative and anti-inflammatory mechanisms results in mitochondrial dysfunction, protein misfolding and altered kinase activity [6]. Post-mortem studies demonstrate presence of intracellular proteinaceous inclusions made of alpha-synuclein in PD brain [7]. Current medications of PD include L-Dopa, dopamine agonists, non-ergot dopamine agonists, ergot-derived dopamine agonists, monoamine oxidase inhibitors and anticholinergics for treatment of early stage motor symptoms in PD. However, treatment of advanced stage motor symptoms includes combinational therapies such as catechol-O-methyltransferase (COMT) inhibitors along with L-DOPA, Monoamine oxidase inhibitors along with L-dopa [8]. Nevertheless, prolonged pharmacological medications cause severe motor (motor fluctuation, dyskinesia) and non-motor (impulse control disorder, PD dementia, sleep disorder) side effects [9]. However, effective disease-modifying therapies for PD is still scarce because the pathological mechanisms of PD are not fully understood. Given that PD genesis is multifactorial, various studies have demonstrated that chronic neuroinflammatory mechanisms play a key role in progressive dopaminergic neuronal loss in PD. Under these conditions, activated microglia and astrocytes release pro-inflammatory factors such as reactive oxygen species (ROS), tumor necrosis factor-α (TNF-α), interleukin-1β (IL-1β), Cyclooxygenase-2 (COX-2), inducible nitric oxide synthase (iNOS) that induce neurodegeneration. Therefore, regulation of glial cells offers potential means of neuroprotection against PD [10]. Recent animal studies demonstrated that rotenone (mitochondrial complex I inhibitor) administration activates microglia and astrocytes resulting in production of pro-inflammatory factors (MMP-9, TNF-α, COX-2, iNOS) and subsequent neuronal death [11]. Similarly, administration of MPP^+^, a mitochondrial complex I inhibitor released MMP-3 which enhanced the expression of TNF-α and ROS production by microglia [12, 13]. In addition to neuroinflammatory mechanisms, alpha-synuclein toxicity is central in PD pathology. Aberrant soluble oligomeric alpha-synuclein species termed protofibrils mediate disturbance in cellular homeostasis and these toxic species have the ability to harbor neighboring cells resulting in disease propagation [14, 15]. Over recent years, experimental evidence demonstrates that autophagy impairment results in accumulation of toxic α-synuclein species and disruption of neuronal homeostasis [16]. Under normal condition, autophagy is activated based on the nutrient need of the cell where misfolded proteins, non-specific long-lived proteins and impaired organelles are degraded to maintain amino acid levels and to prevent apoptosis [17, 18]. However, post-mortem PD samples demonstrated impairment of autophagy-lysosome (ALP) pathways, pathogenic α-synuclein accumulation and mTOR (key regulator of cell growth/proliferation, survival, metabolism, protein synthesis and autophagy) disruption in PD [19, 20].

**Figure 1:**
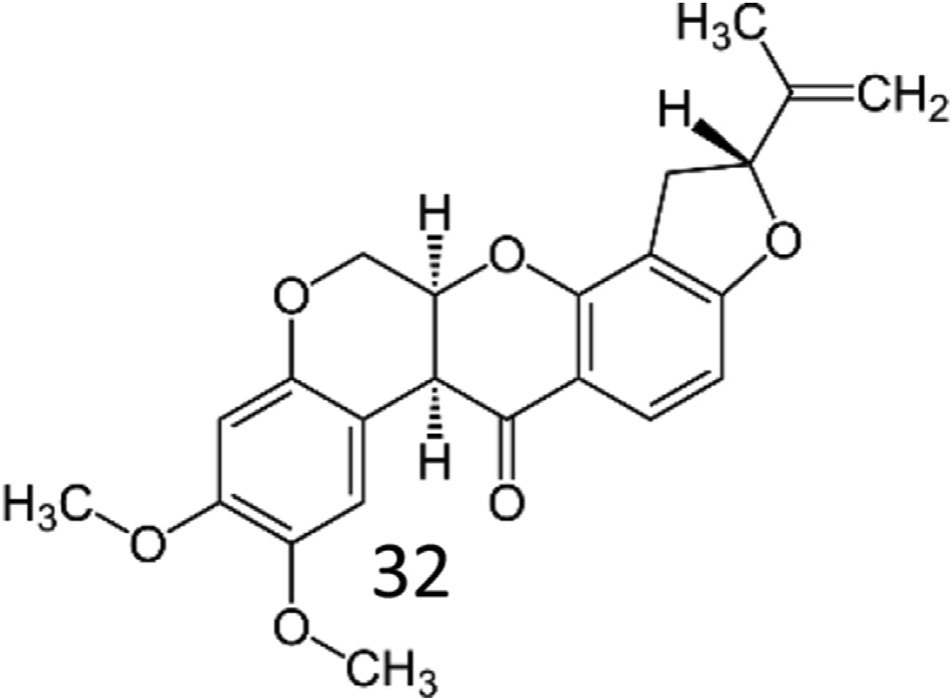
Chemical structure of Rotenone.

**Figure 2:**
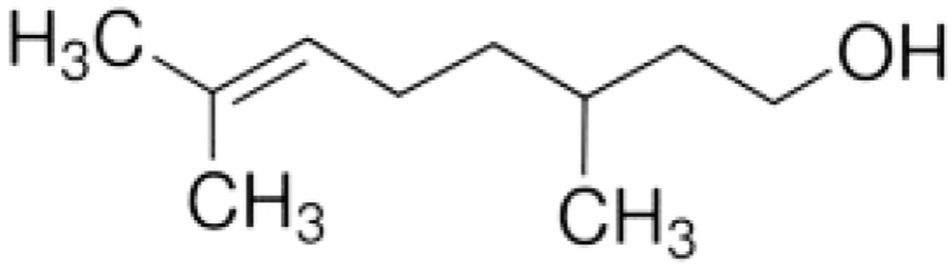
Chemical structure of citronellol.

Rotenone, a mitochondrial electron transport chain complex I inhibitor has a molecular formula of C_23_H_22_O_6_ and molecular weight 394.42. Its chemical structure is shown in Figure 1 causes both behavioral and neuropathological symptoms in rodents as seen in PD. Low rotenone dose cause impairment of calcium signaling, induces oxidative stress and apoptosis, proteasomal dysfunction, loss of tyrosine hydroxylase, nigral iron accumulation and formation of fibrillar cytoplasmic inclusions containing α-synuclein [21]. Unlike MPP+, which requires particular dopamine transporters to cause dopaminergic neuronal death, rotenone crosses the blood–brain barrier due to its high lypophilicity, efficiently causing dopaminergic neuronal death. Rotenone and 6-hydroxydopamine (6-OHDA) also impairs autophagy machinery resulting in α-synuclein accumulation and dopaminergic toxicity, while activation of autophagy protects dopaminergic neurons against rotenone and 6-OHDA toxicity [22]. Hence, rotenone is a widely used animal model to understand the patho-mechanisms of PD. Natural compounds such as phytochemicals have been extensively investigated for their key benefits in treating neurodegenerative diseases. Clinical and experimental studies using phytochemicals provide optimistic results such as enhancement of cognition decreased oxidative stress, neuroinflammation and modulated autophagy pathways [23, 24, 25]. Citronellol, a monoterpene present in *Cymbopogon citrates* and *Cymbopogon winterianus* have been reported to possess hypotensive, analgesic, vasorelaxant, anti-inflammatory and anti-diabetic properties [26, 27, 28]. The chemical structure of Citronellol is shown in Figure 2. Neuroprotective activities such as inhibition of acetylcholine esterase activity, anti-inflammatory and anti-oxidative properties of various compounds isolated from genus citrus have been reported recently [29]. However, knowledge on the effects of citronellol against PD mechanisms is still scarce. Hence, this is the first study to evaluate and understand the neuroprotective mechanisms of citronellol in PD rat model. We have previously demonstrated that rotenone causes oxidative stress, neuroinflammation, enhanced expression of proinflammatory factors, α-synuclein accumulation and dopaminergic neuronal loss in PD rats [11]. Thus, rotenone induced PD rat model was used in this study to understand the mechanisms of citronellol and its efficacy in preventing dopaminergic neuronal death.

## 2 Materials and methods

### 2.1 Drugs and chemicals

Monoclonal mouse anti-α-synuclein was purchased from BD biosciences, San Jose, CA. Polyclonal rabbit anti-cyclooxygenase-2 (Cox-2), anti-inducible nitric oxide synthase (iNOS) and anti-glial fibrillary acidic protein (GFAP) were procured from Sigma-Aldrich, St. Louis, MO, USA. Anti-ionized calcium-binding adaptor molecule-1 (Iba-1) was purchased from Wako chemicals, Richmond, USA. Beta-actin antibody was purchased from Merck Millipore, Darmstad, Germany. Polyclonal rabbit anti-tyrosine hydroxylase antibody was obtained from Merck, Germany Alexa Flour 488 conjugated goat anti-rabbit secondary antibodies were purchased from Thermo Fischer Scientific, Waltham, MA, USA. Biotinylated secondary goat anti-rabbit antibody was purchased from Jackson Immunoresearch, West grove, USA. Citronellol, Rotenone and other analytical grade reagents were procured from Sigma Aldrich, St. Louis, MO, USA. The biochemical assays were performed using commercially-available kits.

### 2.2 Experimental animals and ethics statement

All animal experimental procedures were strictly followed according to the guidelines approved by the Institutional Animal Ethics Committee of the United Arab Emirates University (ERA-2018-5785). In this investigation, adult male Wistar rats weighing 280–300 g (6–7 months old) were employed. Rodents were kept in a pathogen-free environment with ambient temperatures of 22 °C, a 12-hour diurnal cycle, and 60% humidity. Water and food were freely available to the animals. Initially, the animals were given a week to acclimatize to the experimental room conditions.

### 2.3 Experimental design

We employed a validated chronic rotenone PD rat model to investigate citronellol's neuroprotective effects. Rotenone (2.5 mg/kg) was made according previous publication [30]. Citronellol's pharmacological effects in vivo were investigated using the following treatment groups (n = 15/group). Group I received miglyol (i.p.,) and olive oil (oral), which are vehicles for rotenone and citronellol, respectively, and served as the control group in this investigation. The experimental PD model was Group II (rotenone), which received an intraperitoneal injection of rotenone (2.5 mg/kg) once a day for four weeks. Group III (rotenone + citronellol): Citronellol (25 mg/kg, oral) was prepared just before treatment and given once daily for four weeks, 30 min before rotenone. Further, Group IV received citronellol (25 mg/kg, oral) and acted as a drug control group. We used 15 rats/group and observed a mortality rate of 10–15% per group.

### 2.4 Tissue processing

Four weeks after treatment, the animals were anesthetized with pentobarbital (40 mg/kg body weight) and perfused with 0.01 M phosphate-buffered saline at pH 7.4 by intracardial infusion. Tissue samples for biochemical (midbrain) and immunohistochemistry studies were prepared according to previously published protocol [31]. For biochemical studies (n = 6), midbrain and striatum were dissected on dry ice and immediately homogenized using KCl buffer (Tris–HCl 10 mM, NaCl 140 mM, KCl 300 mM, ethylenediaminetetraacetic acid 1 mM, Triton-X 100 0.5%) at pH 8.0 supplemented with protease and phosphatase inhibitor. The homogenates were centrifuged at 14,000× g for 20 min (4 °C) and the supernatant was used for evaluation of lipid peroxidation, antioxidant enzymes and proinflammatory cytokines using spectrophotometric measurements and enzyme-linked immunosorbent assays (ELISA).

### 2.5 Protein estimation

Following the manufacturer's instructions, the Pierce BCA protein assay kit (Thermo Fisher Scientific) was used to determine protein concentration in each sample.

### 2.6 Malondialdehyde assay

The malondialdehyde (MDA) detection kit from North West Life Science (Vancouver, WA, USA) was used to assess the amount of lipid peroxidation in experimental animals. In short, 250 μL of samples or calibrators were incubated with thiobarbituric acid before being vortexed rigorously. The mixture was centrifuged for 3 min at 10,000*g* after 1 h of incubation at 60 °C. The data were presented as m MDA/mg protein and were measured at 532 nm.

### 2.7 Quantification of reduced glutathione

Sigma's glutathione assay kit was used to determine the levels of reduced glutathione in tissue homogenates as per kit's instructions (Sigma-Aldrich Chemie GmbH, Steinheim). In short, deproteinization was done with a 5 percent 5-sulfosalicylic acid solution and the precipitated protein was removed by centrifugation. Reduced glutathione (GSH) was calculated using the supernatant. In 96-well plates, samples or standards (10 μL) were incubated with the working mixture for 5 min. In each well, fifty microliters of diluted Nicotinamide adenine dinucleotide phosphate hydrogen (NADPH) solution was added and thoroughly mixed. Microplate reader was used to measure absorbance at 412 nm with the kinetics for 5 min. Results were expressed as micromolar GSH/mg protein.

### 2.8 Assay for antioxidant enzyme activities

Antioxidant enzyme [superoxide dismutase (SOD) and catalase (CAT)] activities were assessed using Cayman assay kits (Cayman Chemicals Company, Ann Arbor, MI, USA) in experimental animals. Superoxide dismutase assay: In 96-well plates, ten microliters of samples or standards were added. To initiate the process, 20 μL of xanthine oxidase was added. The solution was briefly mixed before being incubated (covered) at room temperature for 30 min. A microplate reader was used to measure absorbance at 450 nm. SOD activity was measured in units per milligram of protein. Catalase assay: Twenty microliters of samples or standards were added to the assay buffer (100 μL) in 96-well plates, along with 30 μL of methanol. To initiate the process, H_**2**_O_**2**_ was added to the mixture and incubated for 20 min at room temperature (RT). After incubation, the process was stopped with 30 μL of potassium hydroxide. Then 30 μL of purpald catalase and 10 μL of catalase potassium periodate were added. The plate was incubated in a shaker for 5 min at room temperature before being measured at 540 nm with a microplate reader. Catalase activity was measured in nanomoles per minute per milligram of protein.

### 2.9 Proinflammatory cytokines and MMP-9 ELISA assay

Anti-inflammatory activity of citronellol was analyzed using commercially available ELISA kits from R&D systems, Minnesota, United States (IL-6: Cat. No. DY506, TNF-α: Cat. No. DY510, IL-1β: Cat. No. DY501). 100 μL of capture antibody (diluted) was used to coat ninety-six well plates and the plates were incubated overnight at RT. After incubation, each well was rinsed with wash buffer and blocked with blocking buffer for 1 h. After washing the plates, 100 μL of samples or standards were added and incubated for 2 h at room temperature. The plate was then incubated at RT for 2 h with 100 μL of detection antibody in each well. After incubation, the plates were rinsed and 100 μL of working solution was added, followed by 20 min of incubation. Later, the wells were replaced with 100 μL of substrate solution, and the plate was incubated for another 20 min. Finally, the stop solution was added, and the contents of the plate were gently mixed. A microplate reader was used to read the absorbance instantly at 450nm. Our results were expressed in terms of pg/mg protein.

### 2.10 Assessment of microglia and astrocyte activation by immunofluorescence staining

The anti-inflammatory activity of citronellol in experimental animals (n = 5) was studied using immunofluorescence staining. The striatum sections (20 μm) were washed twice with PBS and blocked for 1 h at room temperature. Anti-rabbit Ionized calcium binding adaptor molecule 1 (Iba-1, 1:1000, Wako chemicals, Richmond, USA) and anti-rabbit glial fibrillary acidic protein (GFAP, 1:1000, Sigma-Aldrich, St. Louis, MO, USA) antibodies were incubated at 4 °C overnight after the blocking reagent was removed. The sections were then washed twice with PBS before being incubated for 1 h at room temperature with the corresponding fluorescent secondary antibody (Alexa 488 anti-rabbit, Thermo Fischer Scientific, Waltham, MA, USA). The stained slices were rinsed in PBS twice before being mounted with anti-fade mounting media. Images were taken with a Nikon Eclipse Ni fluorescence microscope.

### 2.11 Quantification of activated astrocytes and microglia in the striatum

Activated astrocytes and microglia were examined in three stained coronal slices of the same level of striatum from each brain. The activation of microglia and astrocytes was measured using a previously reported method [31]. Three fields of equal area were picked at random and evaluated with ImageJ software (NIH, Bethesda, MD, USA). Briefly, the fluorescence intensity, area and circularity were measured by drawing an outline on the area of interest. Background readings were measured in this similar way. The following equation was used to obtain total corrected cellular fluorescence (TCCF), TCCF = integrated density − (area of selected cell × mean fluorescence of background readings) [32]. To prevent bias, fluorescence intensity was measured by an observer blind to the experimental groups. Results were expressed as percentage of control.

### 2.12 Immunoblot analysis

Using a previously described protocol [31], the expression of α-synuclein, COX-2, iNOS, Bax, Bcl-2, mTOR, and p62 in the midbrain of experimental animals (n = 3) was evaluated. Briefly, tissues were homogenized in RIPA buffer (R0278, Sigma Aldrich, St. Louis, MO, USA) supplemented with protease and phosphatase inhibitors and centrifuged for 20 min at 12,000 rpm. On SDS-PAGE, an equal amount of protein (20 μg) from each sample was loaded and separated. By using a semi-dry transfer approach, the separated proteins were electrotransferred onto a PVDF membrane (BIO-RAD). The membranes were incubated overnight at 4 °C with Nrf2 (1:750, Abcam, USA), α-synuclein (1:750, BD Biosciences, San Jose, CA), COX-2 (1:2000, Sigma-Aldrich), iNOS (1:1000, Sigma-Aldrich), Bax (1:2000, Abcam, USA), Bcl-2 (1:500, Abcam, USA), mTOR (1:1500, Cell Signalling Technology, Beverly, MA, USA), p62 (1:900, Cell Signalling Technology, Beverly, MA, USA) after blocking (1 h in 5 percent nonfat dry milk in TBS at RT). The membranes were washed after incubation and horseradish peroxidase conjugated secondary antibodies were added. After 1 h, the membranes were washed and Enhanced Chemiluminescence Pico Kit (Thermo Fisher Scientific, USA) was used to visualize the bands. The generated blots were stripped and reprobed for β-actin (1:5000) as a loading control, followed by densitometry analysis with “ImageJ” analysis software.

### 2.13 Immunohistochemistry analysis

As previously disclosed, cryoprotected animal (n = 5) brains were serially sectioned (20 μm) using a cryostat (Leica, Wetzlar, Germany). To inactivate tissue peroxidase, 1 percent hydrogen peroxidase (in PBS) was applied to the sections after they had been washed twice. After two washes with PBS, the sections were blocked for 30 min at RT with blocking reagent before being incubated overnight at 4 °C with goat anti-rabbit tyrosine hydroxylase (TH) polyclonal antibody (1:1000, Merck, Germany). After that, the sections were washed twice with PBS and incubated for 1 h at room temperature with biotinylated secondary anti-rabbit (1:1000) antibody. Following incubation, sections were developed with the avidin-biotin peroxidase complex system (ABC kit, Vectastain, USA) and 3,3′ diaminobenzidine (DAB). After staining, the sections were coverslipped with DPX mounting solution and imaged under a light microscope (Olympus, Hamburg, Germany).

### 2.14 Assessment of TH-ir dopaminergic neurons and TH-ir dopamine nerve fibers loss

Dopaminergic neuron loss in the SNpc was investigated by counting neurons at three distinct levels of the medial terminal nucleus area. The average value was expressed as percentage of control [31]. ImageJ software was used to assess TH-ir dopaminergic fibers optical density in the striatum. Three different fields per section with equal area within the striatum was used to measure the optical density of TH-ir fibers. The average of three areas were calculated and reported as percentage of control. Background measure was obtained by assessing the optical density of the overlying cortex and subtracted from the value generated from the striatum. To prevent bias, counting TH-ir neurons and measuring the OD of TH-ir fibers in striatum was done by an observer blind to the experimental groups.

### 2.15 Statistics

The data were presented as the mean value SEM. Using the SPSS 12 program, a one-way ANOVA followed by a Tukey's test was used to determine the statistical significance of different treatment groups. P < 0.05 was declared statistically significant.

## 3 Results

### 3.1 Citronellol diminished rotenone induced oxidative stress

**Figure 3:**
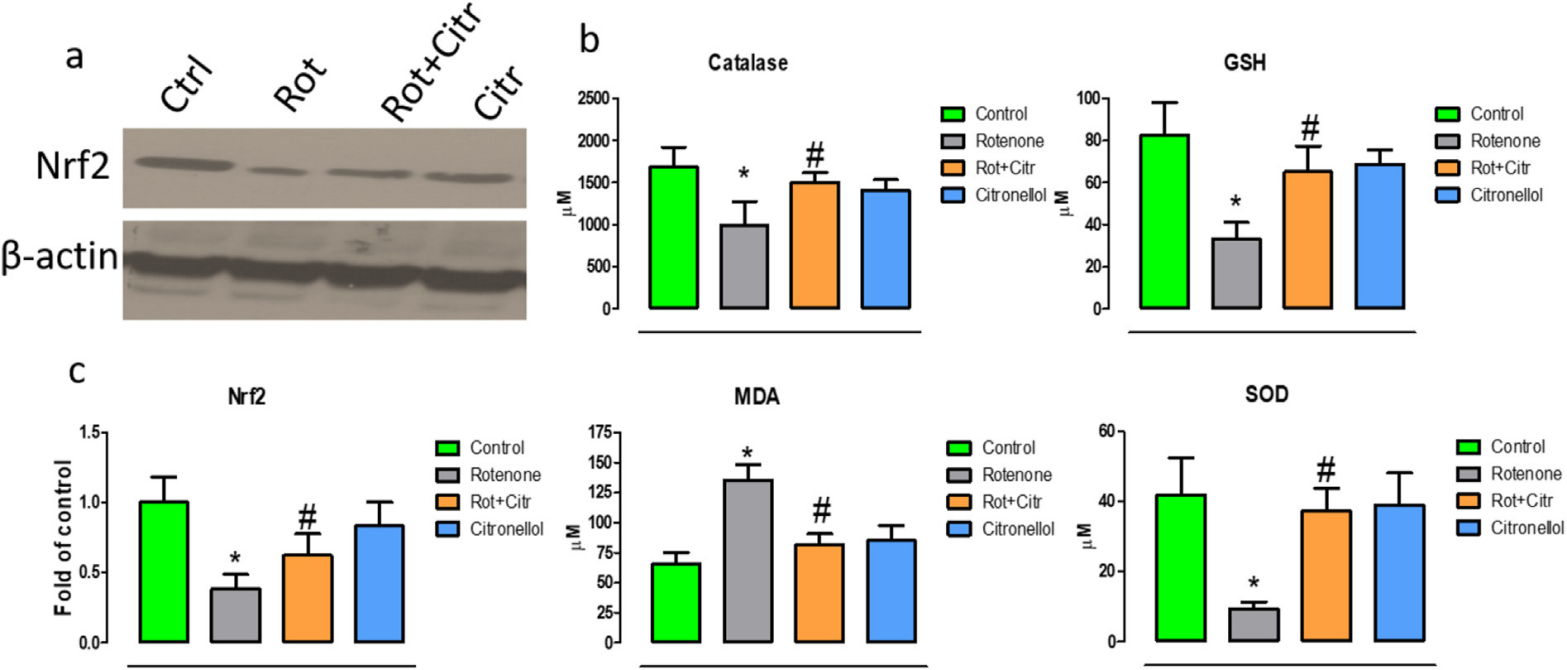
Citronellol prevented rotenone induced oxidative stress. Effect of citronellol on Nrf2 expression and antioxidant enzymes have been measured in midbrain of experimental animals. Antioxidant activities in experimental samples (midbrain) were measured using commercially available kits. Rotenone administration (2.5 mg/kg, i.p) significantly increased lipid peroxidation and decreased Nrf2 expression (a) and antioxidant levels (b) in the midbrain. However, oral administration of citronellol (25 mg/kg) reduced lipid peroxidation levels and significantly reverted antioxidant (CAT, GSH and SOD) levels. Quantitative evaluations of blots were done using ImageJ and their results were represented in histogram (c). Data are represented as mean ± SD. ∗p < 0.05 compared to control, ^#^p < 0.05 compared to rotenone alone treated group.

Our study showed that rotenone administration significantly decreased expression of Nrf2 (*F*_3,20_ = 17.94, p < 0.001), a master antioxidant regulator that resulted in reduction in vital antioxidants such catalase (*F*_3,20_ = 6.51, p = 0.002), glutathione (*F*_3,20_ = 21.07, p < 0.001)and superoxide dismutase (*F*_3,20_ = 22.98, p < 0.001). In addition, malondialdehyde (MDA) levels, a marker of lipid peroxidation were increased (*F*_3,20_ = 44.50, p < 0.001). Conversely, administration of citronellol 30 min prior to rotenone exposure reversed Nrf2 expression and enhanced antioxidant activity along with significant decrease in MDA levels (Figure 3). These results showed that citronellol diminished free radical production through its anti-oxidative properties.

### 3.2 Citronellol treatment prevented rotenone induced neuroinflammation

**Figure 4:**
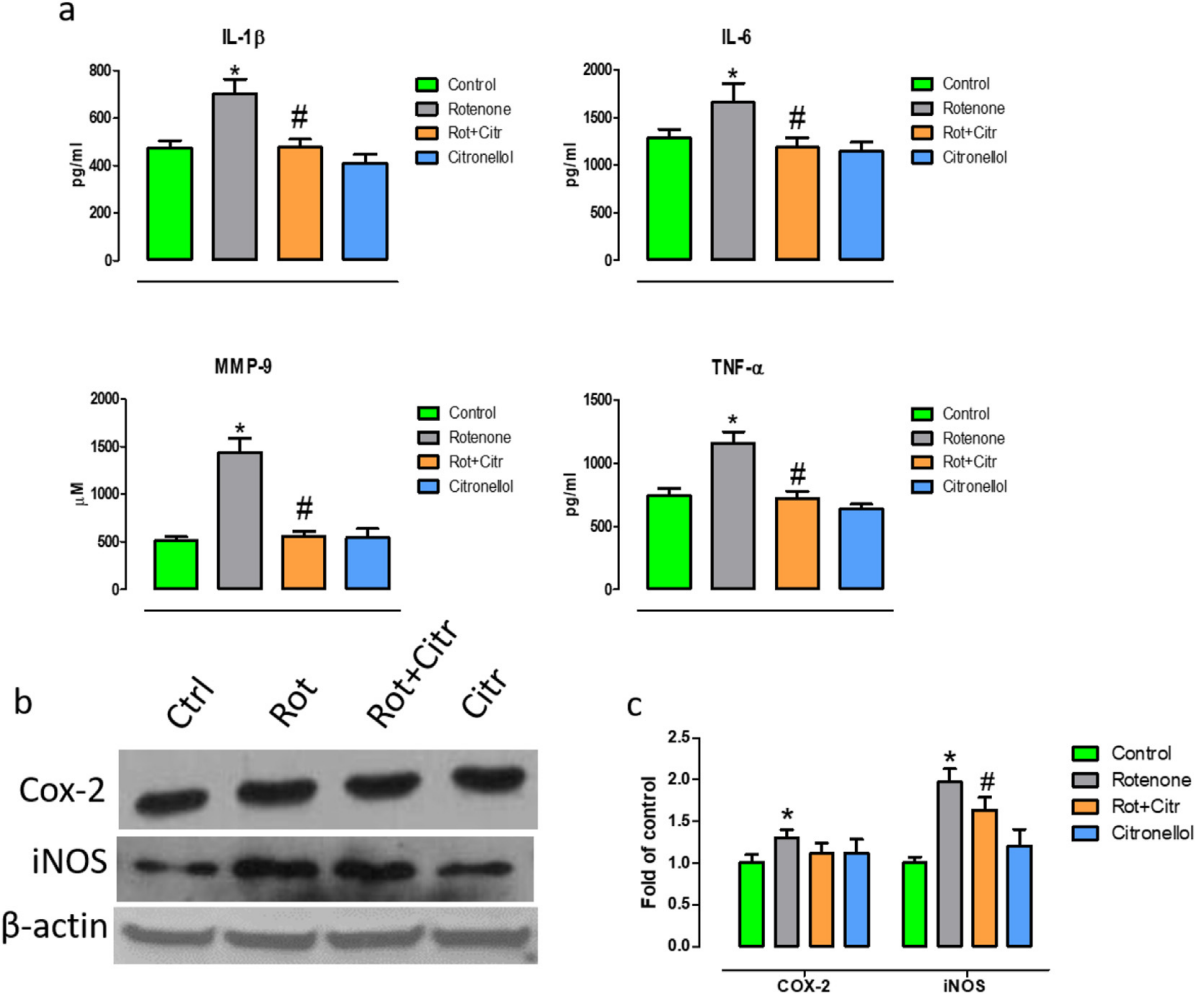
Citronellol prevented production of pro-inflammatory factors in experimental animals. ELISA and western blotting were performed to analyze the effect of citronellol on pro-inflammatory markers in the midbrain of experimental animals. Administration of rotenone (2.5 mg/kg, i. p) for four weeks significantly enhanced secretion of pro-inflammatory cytokines such as IL-1β, IL-6, TNF-α and MMP-9. (a). Further, rotenone enhanced the expression of COX-2 and iNOS in midbrain of experimental animals (b). Quantitative evaluations of blots were done using ImageJ and their results were represented in histogram (c). However, administration of citronellol (25 mg/kg, oral) for four weeks significantly decreased the expression of pro-inflammatory factors. Data are represented as mean ± SD. ∗p < 0.05 compared to control, ^#^p < 0.05 compared to rotenone alone treated group.

Further, to understand the anti-inflamamtory mechanisms of citronellol, we evaluated the production of pro-inflammatory factors that augment apoptotic mechanisms. We found that administration of rotenone significantly increased secretion of pro-inflammatory factors such as interleukin-6 (IL-6) (*F*_3,20_ = 19.30, *p* < 0.001), interleukin-1β (IL-1β) (*F*_3,20_ = 54.47, *p* < 0.001), tumor necrosis factor-α (TNF-α) (*F*_3,20_ = 78.37, *p* < 0.001) and matrix metalloproteinase 9 (MMP-9) (*F*_3,20_ = 140.78, *p* < 0.001) levels (Figure 4a). Nonetheless, administration of citronellol diminished IL-6, IL-1β, TNF-α and MMP-9 secretion. Further, rotenone intoxicated rats had higher expression of iNOS (*F*_3,20_ = 15.28, *p* < 0.001) and COX-2 (*F*_1,10_ = 30.60, *p* < 0.001 vs Control), two critical inducible enzymes responsible for pro-inflammatory mechanisms [33, 34]. However, administration of citronellol diminished the expression of COX-2 and iNOS as evident from western blotting (Figure 4c,d).

### 3.3 Citronellol reduced microglia and astrocyte activation

**Figure 5:**
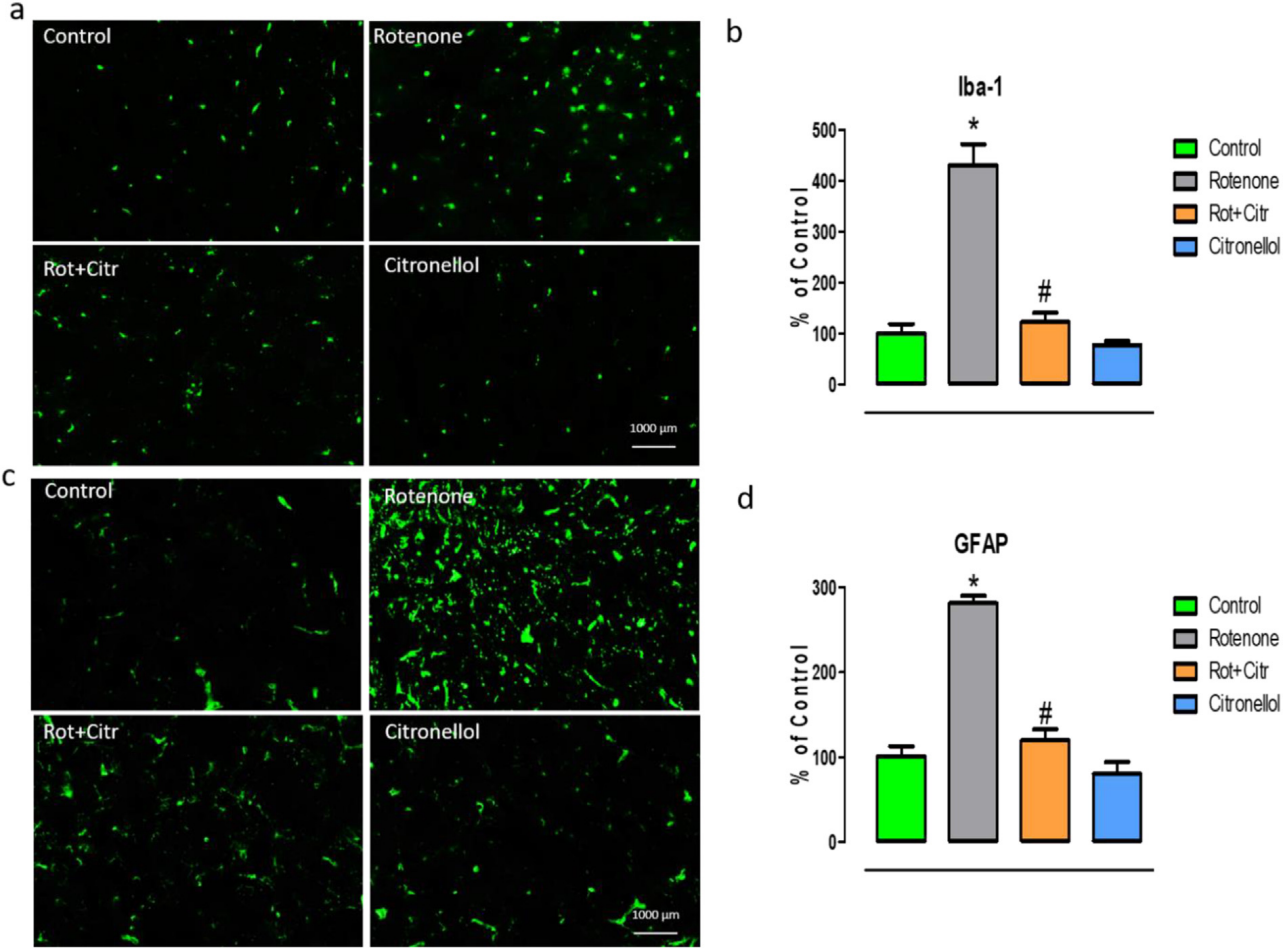
Citronellol reduced microglia and astrocytes activation. Immunofluorescent staining for microglia and astrocyte was performed to understand the anti-inflammatory property of citronellol. Over expression of Iba-1 and GFAP is an indicator of activation of microglia and astrocyte respectively. Our studies showed that rotenone administration (2.5 mg/kg, i.p) activated microglia as evident from larger cell bodies and higher fluorescence intensity (a). Similarly, higher expression of GFAP represents activation of astrocytes (c). However, citronellol administration (25 mg/kg, oral) decreased the activation of microglia and astrocytes. ImageJ was used to quantify the activation of microglia and astrocytes. Results are represented as percentage of control in the corresponding histograms (b and d). Data are expressed as mean ± SD. ∗p < 0.05 compared to control, ^#^p < 0.05 compared to rotenone alone treated group.

Higher expression of Iba-1 and GFAP reciprocates the activation of microglia and astrocyte respectively. Our immunofluorescent results demonstrate the activation of microglia and astrocytes in the striatum of rotenone treated animals. Larger cell bodies and fewer processes and enhanced expression of Iba-1 (*F*_3,8_ = 138.43, *p* < 0.001) represent activated microglia (Figure 5a). Similarly, enhanced expression of GFAP (*F*_3,8_ = 167.23, *p* < 0.001) is a typical marker for astrocytes activation (Figure 5c). Interestingly, administration of citronellol significantly diminished activation of microglia and astrocytes in experimental animals. Activation of microglia and astrocyte were quantified using ImageJ and their values are represented as percentage of control in corresponding histograms (Figures 5b and 5d).

### 3.4 Citronellol prevented dopaminergic neuronal loss

**Figure 6:**
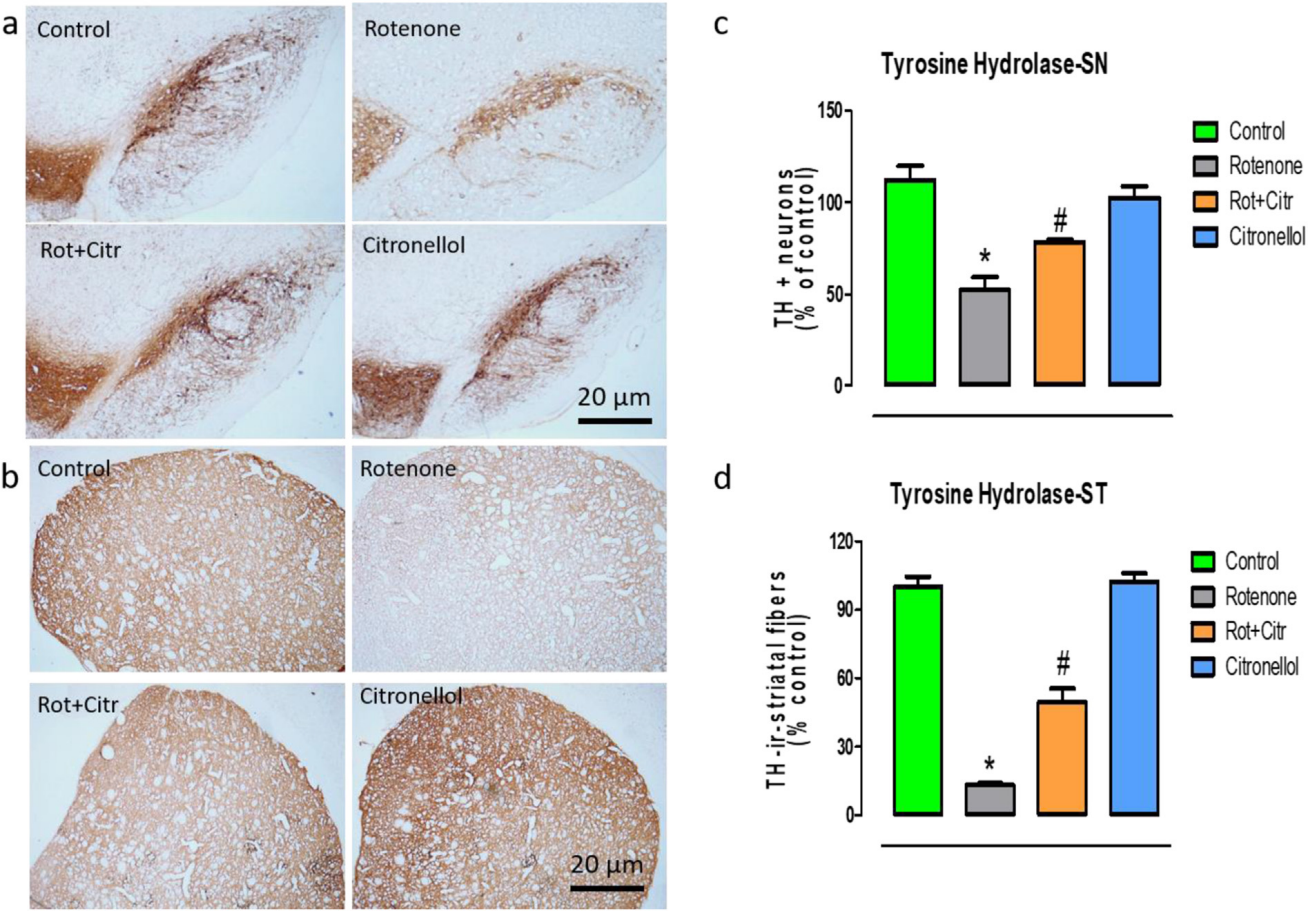
Citronellol diminished rotenone induced dopaminergic neuronal loss. Post-mortem studies clearly demonstrated loss of tyrosine hydroxylase (TH) positive dopaminergic neurons in the SNpc with significant decrease in the expression of tyrosine hydroxylase in the striatum. Cryoprotected animal brains were serially sectioned (20 μm) using a cryostat and tyrosine hydroxylase immunohistochemistry was performed. Our results demonstrated significant decrease in number of TH positive dopaminergic neurons (a, b) with concomitant decrease in the intensity of TH + ve striatal fibers (c, d) in rotenone intoxicated animals. However, citronellol administration reduced dopaminergic neuronal loss and preserved the expression of tyrosine hydroxylase in striatal fibers. Data are expressed as mean ± SD. ∗p < 0.05 compared to control, ^#^p < 0.05 compared to rotenone alone treated group.

Reduced tyrosine hydroxylase expression leads to less dopamine synthesis which results in PD. Hence, we quantified TH + ve dopaminergic neurons in SNpc and its expression in the striatum. Our results showed that administration of rotenone significantly reduced the number of TH positive neurons (*F*_3,8_ = 56.51, *p* < 0.001) (Figure 6a,b) with significant loss of TH + ve (*F*_3,8_ = 319.92, *p* < 0.001)striatal fibers (Figure 6c,d). Our results were in concordant with earlier published report [11]. However, administration of citronellol significantly reduced dopaminergic neuronal loss and preserved the expression of tyrosine hydroxylase in the striatal fibers.

### 3.5 Citronellol prevented over-expression of α-synuclein in rotenone intoxicated rats

**Figure 7:**
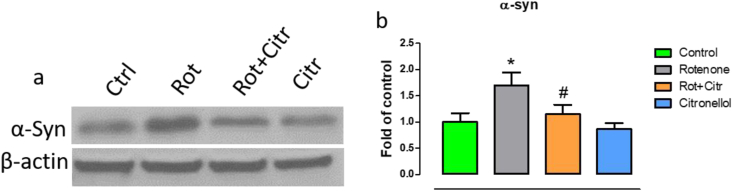
Citronellol prevents α-synuclein over-expression in rotenone animals. Immunoblots representing alpha-synuclein expression in midbrain of experimental animals (a). Blots were quantified using ImageJ and the results are represented as histogram (b). Results are represented as mean ± SD. ∗p < 0.001 compared to control, ^#^p < 0.001 compared to rotenone treated group.

Over expression of alpha-synuclein results in lewy body formation, activation of caspases and dopaminergic neuronal loss in PD [35]. Thus, we evaluated the expression of α-synuclein in the midbrain of our experimental animals. Western blotting results demonstrated that rotenone administration significantly (*F*_3,20_ = 8.65, *p* < 0.001) increased α-synuclein expression (Figure 7). However, citronellol reduced the expression of α-synuclein significantly.

### 3.6 Citronellol protected dopaminergic neurons by modulating apoptosis and restoring mTOR pathway

**Figure 8:**
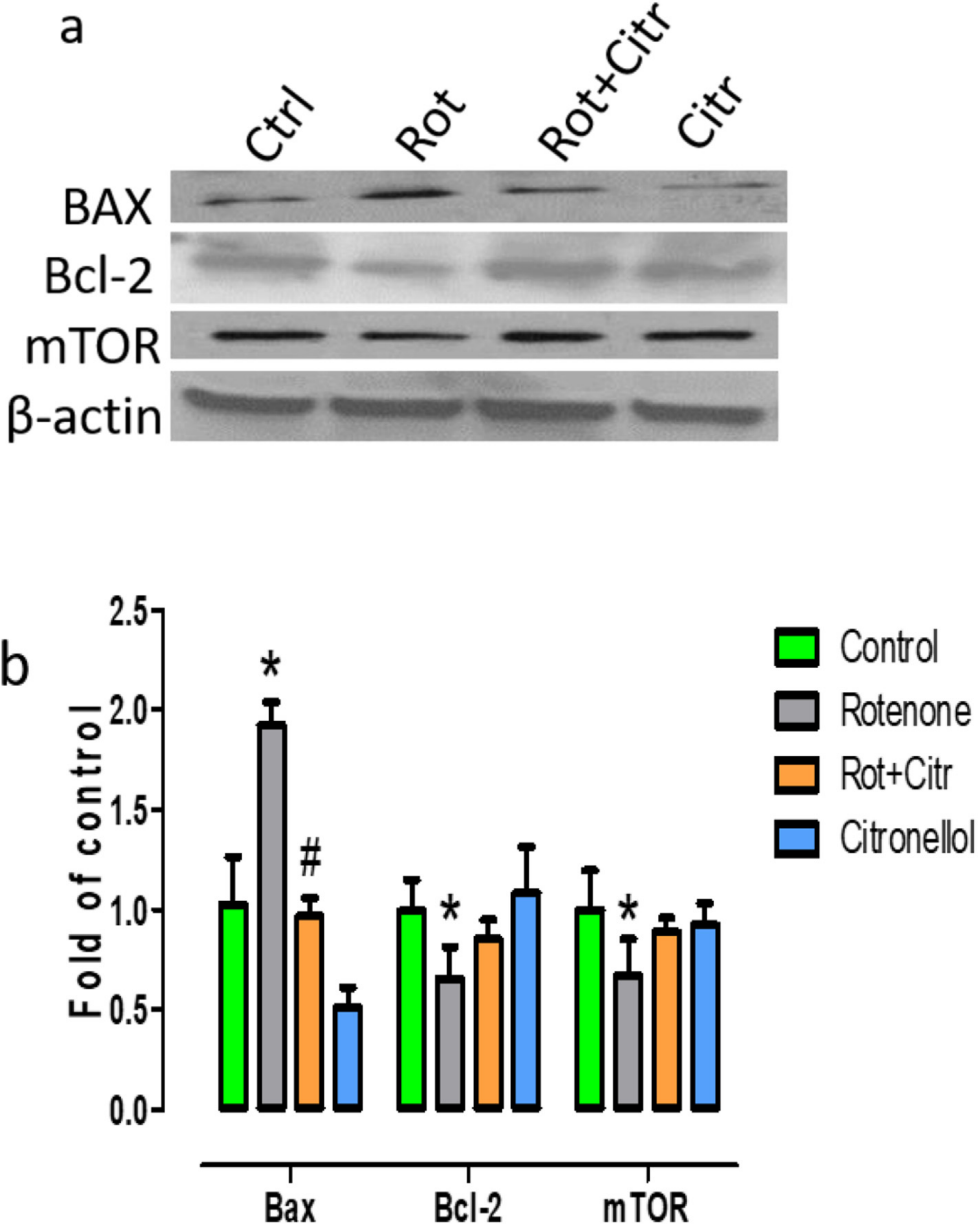
Citronellol prevented apoptosis and enhanced mTOR expression. Western blot analysis was done to examine the expression of Bax, Bcl-2 and mTOR in the midbrain of experimental samples (a). Results were quantified using ImageJ and represented as fold of control (b). Results are expressed as mean ± SD. mTOR: ∗p = 0.037 compared to control, Bcl-2: ∗p = 0.001 compared to control, Bax: ∗p < 0.05 compared to control, ^#^p < 0.05 compared to rotenone alone treated group.

Disruption of mTOR signaling impairs neuronal function and favors apoptosis and neurodegenerative mechanisms [36, 37]. Immunoblot analysis of midbrain samples showed that mTOR expression was significantly (*F*_1,10_ = 5.73, *p* = 0.037 vs Control) reduced in rotenone intoxicated rats. In addition, we evaluated whether reduced mTOR expression increased apoptosis. We found that rotenone significantly enhanced the expression of pro-apoptotic marker Bax (*F*_3,20_ = 97.31, *p* < 0.001) and decreased the expression of anti-apoptotic protein Bcl-2 (*F*_1,10_ = 13.90, *p* = 0.003 vs Control). However, oral administration of citronellol prevented apoptosis and restored mTOR expression (Figure 8).

### 3.7 Citronellol prevented dopaminergic neurons loss through regulating autophagy

**Figure 9:**
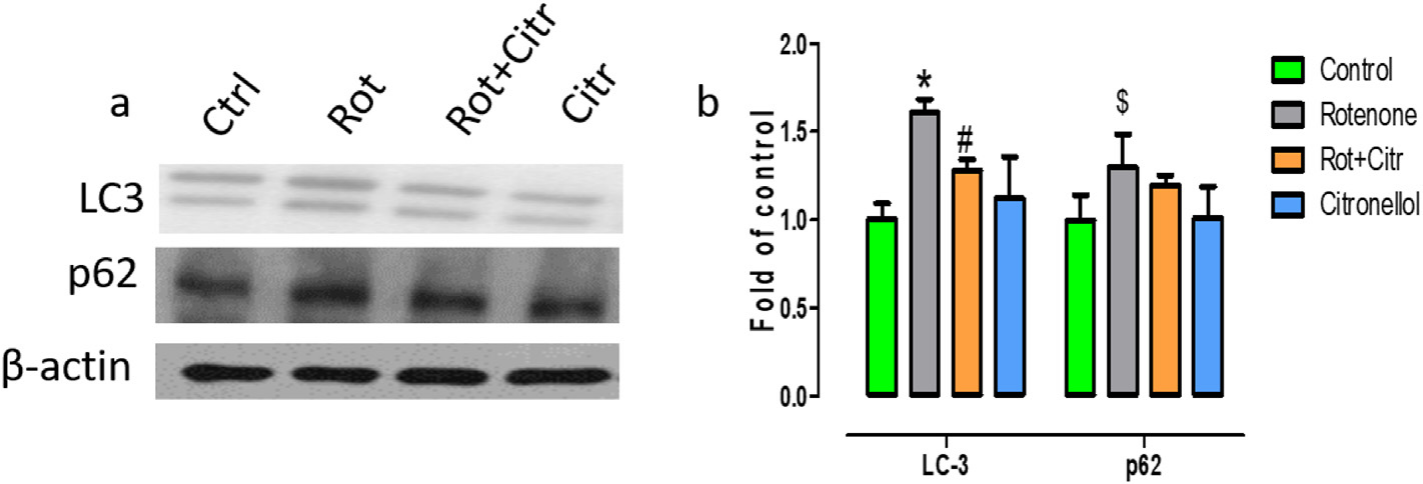
Citronellol protected dopaminergic neurons by restoring autophagy pathway. Midbrain levels of autophagy markers LC3 and p62 were evaluated by western blotting (a). Quantitative evaluation of LC3 and p62 levels were done using ImageJ (b). Values are represented as mean ± SD. ∗p < 0.05 compared to control, ^#^p < 0.05 compared to rotenone alone treated group, ^$^p = 0.05 compared to control.

Alpha-synuclein aggregation and autophagy dysfunction play a major role in dopaminergic neuronal loss in PD. Thus, we wanted to evaluate whether citronellol modulated autophagy pathway by studying the expression of MAP-light chain 3 (LC3) and p62 expression levels Administration of rotenone increased LC3 (*F*_3,20_ = 7.71, *p* = 0.001) expression, a marker for autophagosome accumulation. In addition, rotenone administration significantly enhanced the expression of autophagic substrate p62 (*F*_3,20_ = 2.93, *p* = 0.05). However, citronellol administration decreased both LC3 and p62 levels favoring autophagic degradation of misfolded proteins (Figure 9).

## 4 Discussion

Taken together, we report for the first time that citronellol, a monoterpene found in essential oils, protected dopaminergic neurons from rotenone neurotoxicity through its anti-oxidant, anti-inflammatory, anti-apoptotic and autophagy modulating properties.

Rotenone, MPTP, and 6-OHDA have all been utilized extensively in animal models to better understand the pathogenic underpinnings of Parkinson's disease. Rotenone, a classic mitochondrial complex I inhibitor, is frequently utilized to better understand the pathogenic underpinnings of Parkinson's disease and to assess the efficacy of new medication candidates in treating the disease [38, 39, 40]. As a highly lipophilic PD mimetic that, unlike other PD mimics such as 1-methyl,4-phenyl,1,2,3,6-tetrahydropyridine (MPTP), rotenone does not require dopamine receptors to pass the blood-brain barrier, making it a useful tool for studying lewy-body development too [41]. Apart, it degenerates dopaminergic neurons by enhancing oxidative stress, neuroinflammation, α-synuclein aggregation, autophagy impairment, dopaminergic neurodegeneration and resultant behavioral deficits [11, 42]. Thus, we evaluated the neuroprotective mechanism of citronellol using rotenone induced PD model.

Given the proven safety of herbal drugs and the side-effects of current medications, various clinical studies are being conducted to analyze the effect of phytochemicals on PD [22]. Phytochemicals rich in antioxidants maintains neuronal integrity through neuroprotection, neuroplasticity and neurorestoration [43, 44]. Dopaminergic neurons are extremely susceptible to oxidative stress due to high metabolic rates along with highly oxidizable iron and polyunsaturated fatty acids species. Though various mechanisms are involved in cause of dopaminergic neuronal, oxidative stress acts as a critical mechanism consistently in all the PD models [45]. Supporting this evidence, various studies reported impairment of mitochondrial complex I mechanisms [46], oxidation/nitration of proteins and iron [47, 48], reduction in Nrf2 expression and antioxidants levels [49]. Hence, we analyzed the ability of citronellol to modulate Nrf2 expression and antioxidant levels in rotenone treated rats. Citronellol, a acyclic and monocyclic monoterpenes present in *Citrus hystrix* possess antioxidant and acetylcholinesterase inhibition activities [50]. Defensive antioxidant enzymes (GSH, SOD and catalase) neutralizing ROS functions are produced as protective mechanisms in the brain. Rotenone intoxication causes mitochondrial dysfunction resulting in production of free radicals and oxidative stress. Our results demonstrated that rotenone increased oxidative stress. However, administration of citronellol significantly enhanced Nrf2 expression and antioxidant enzyme (GSH, SOD and catalase) levels and prevented lipid peroxidation/oxidative stress in rotenone treated rats. Further, Lee et al., demonstrated that rotenone treated Nrf2^−/−^ neurons were more susceptible to apoptosis compared to controls [51]. The ability of citronellol to scavenge free radicals might be due to the reducing power of the compound. Our results were supported by experiments demonstrating DPPH free radical scavenging activity and ferric reducing properties of citronellol [52]. Under physiological conditions, monoamine oxidase, a mitochondrial bound isoenzyme, catalyzes oxidative deamination of various neurotransmitters including dopamine, which results in increase in hydrogen peroxide levels. However, during early PD, higher dopamine turnover may generate higher hydrogen peroxide with subsequent hydroxyl radical production resulting in dopaminergic neuronal death [53]. Hence, citronellol could be developed as a potential therapeutic agent due to its antioxidant properties demonstrated in the current study.

In addition to oxidative stress, neuroinflammation is one of the common denominators of PD [54]. Unlike acute neuroinflammatory response that are beneficial, chronic neuroinflammation exacerbates PD progression. Various clinical studies demonstrated that inflammation worsens neurodegeneration in PD [55]. Microglia and astrocytes are major inflammatory cells involved in neuroinflammation-associated neurodegeneration. These activated glial cells release proinflammatory factors that fuel neurodegeneration. Similar to previous reports, we demonstrate that rotenone induced oxidative stress activated microglia (Iba-1 staining) and astrocyte (GFAP staining) resulting in release of pro-inflammatory factors such as IL-6, IL-1β and TNF-α, MMP-9, COX-2 and iNOS [56, 57]. However, administration of citronellol reverted this toxic response. Autopsy studies have also demonstrated higher expression of pro-inflammatory factors in PD brains [58, 59, 60, 61]. Supporting our results, previous studies have reported that citronellol reduced nitric oxide and prostaglandin E2 levels in LPS treated macrophages and diminished COX-2 expression [62, 63]. In addition, microglial interaction with apoptotic neurons promotes COX-2 expression and PGE synthesis [64]. Natural compound have been demonstrated to decrease neuroinflammation by modulating Nrf2-Responsive Antioxidant Element (ARE) pathway. Hence, anti-inflammatory effect of citronellol can be attributed to the antioxidant potential and Nrf2 modulating properties of citronellol.

Alpha-synuclein, a 14 kDa synaptic protein enhances neuroinflammation and they are abundant in pre-synaptic terminals, a major hotspot for aggregation. Post-mortem studies reported higher alpha-synuclein levels in lewy bodies of PD patients. In addition, wild type or mutant forms of α-synuclein results in apoptosis and enhances neuronal sensitivity to apoptotic death [65, 66]. Therefore, we wanted to analyze the effect of citronellol on α-synuclein expression. Citronellol administration significantly decreased α-synuclein expression compared to rotenone groups. Animal studies have demonstrated that α-synuclein over-expression activates microglia with higher secretion of pro-inflammatory factors [67]. Hence, ability of citronellol to decrease alpha-synuclein expression could in turn prevent microglia and astrocyte activation and resultant inflammatory response. This is the first study demonstrating the effect of citronellol on α-synuclein expression. However, geraniol, a monoterpene with close structural similarity to citronellol decreased α-synuclein expression and protected dopaminergic neurons in MPTP induced PD model [68]. Further, to evaluate whether these properties of citronellol could protect dopaminergic neurons from apoptosis, we analyzed tyrosine hydroxylase expression in substantia nigra and striatum of experimental animals. Tyrosine hydroxylase, a rate-limiting enzyme for dopamine formation, is usually studied to access survival of dopaminergic neurons. As evident from previous reports, we demonstrated that rotenone significantly reduced dopaminergic neurons in SN along with reduced tyrosine hydroxylase expression in striatum [30, 31]. Conversely, citronellol protected dopaminergic neuronal and prevented striatal fiber loss which might be due to its anti-oxidative and anti-inflammatory properties. Further, to understand the molecular mechanisms involved in neuroprotection, we evaluated expression of proteins involved in apoptotic and autophagy pathways.

Apoptosis, a major programmed cell death pathway is initiated by various stimuli such as stress, activation of death receptors, excitotoxicity, afferent or efferent trophic factor deprivation. In normal physiological state, apoptosis is crucial for neural circuit development and shaping of nervous system [69]. Various morphological and biological changes such as cell shrinkage, chromatin condensation, DNA fragmentation and development of apoptotic bodies are hallmark event involved in apoptosis. However, in PD, apoptosis is reported to be a crucial neuronal death mechanism due to the presence of DNA fragmentation, apoptotic chromatin changes and over-expression of caspase-3 in dopaminergic neurons of PD patients [70, 71]. Intrinsic apoptotic pathway results in cytochrome c release into the cytosol followed by permeabilization of membrane by Bax and decrease in anti-apoptotic protein Bcl-2. These events results in reduced ATP synthesis and eventually cell death. Further bridging the connection between apoptosis and α-synuclein in PD, over-expression of alpha-synuclein reduces anti-apoptotic protein Bcl-2 [72]. Under normal conditions, apoptosis is crucially governed by mTOR signaling that plays a major role in cell shape maintenance, migration and differentiation during neuronal development [73]. Under pathological conditions like PD, mTOR impairment leads to aggravation of oxidative stress pathways and apoptotis [74]. Administration of rotenone enhances apoptosis by suppressing mTOR cascade suggesting the neuroprotective significance of mTOR pathway [75]. Similar to these reports, we found that rotenone diminished mTOR expression and enhanced apoptosis by increasing the expression of Bax and decreasing the expression of anti-apoptotic Bcl-2. However, administration of citronellol prevented these changes significantly. Our results suggest that the anti-apoptotic property of citronellol could have played a major role in protecting dopaminergic neurons against rotenone toxicity.

Autophagy, a crucial catabolic mechanism involved in degradation of unwanted/misfolded proteins plays a crucial role in PD. Excessive apoptosis and autophagic disturbance is strongly linked with PD. Post-mortem and experimental studies demonstrate that build-up of autophagic vacuoles (identified by LC3 levels) is toxic to dopaminergic neurons [72, 76, 77]. Under normal conditions, heat shock cognate 70 (HSC70) chaperone protein binds to α-synuclein by identifying the pentapeptide sequence motif [78]. Later, α-synuclein binds to lysosomal-associated membrane protein type 2A (LAMP-2A) at the lysosomal membrane where it is degraded by proteases. In PD, buildup of autophagic vacuoles is associated with less lysosomal degradation which is demonstrated by increase in p62 levels (autophagy substrate) [79]. Similar to these reports, we found that rotenone induced PD animals had significantly higher LC3 expression, a marker for autophagic vacuole accumulation. However, autophagic vacuole accumulation might be due to less lysosomal degradation or due to impairment of autophagy induction. Hence, we assessed p62 levels, a marker for autophagic flux in the experimental animals. P62 is an ubiquitinating and LC3 binding protein that binds with aggregated proteins resulting in autophagy degradation. Rotenone administration enhanced p62 levels indicating that rotenone blocks autophagic vacuole degradation [80]. Alternatively, we found that citronellol decreased p62 levels although this was not significant. Supporting our results, Wu et al., demonstrated that rotenone significantly increased LC3 and p62 levels in rotenone rat model [81]. Although this is the first study to evaluate autophagy modulating property of citronellol, geraniol, an acyclic monoterpene derived from citronella was previously reported to decrease LC3 levels resulting in autophagy clearance dependent neuroprotection [82].

## 5 Conclusions

Taken together, our data suggests that citronellol protects dopaminergic neurons by (i) restoring antioxidant defense mechanisms and decreasing rotenone induced oxidative stress (ii) suppressed neuroinflammation by decreasing microglia and astrocytes activation with subsequent decrease in pro-inflammatory factors secretion (iii) prevented apoptosis (iv) modulated autophagy by inhibiting autophagy vacuole accumulation and promoted lysosomal degradation of α-synuclein. In view of the need of medications for PD with fewer side effects and which has the ability to halt disease progression, we would like to highlight terpenes such as citronellol could be developed as an supplement for PD. However, this is an initial study and more experiments on different animal models are crucial to understand the pro-survival mechanisms of citronellol in preventing neurodegeneration.

## Declarations

### Author contribution statement

Richard L. Jayaraj: Performed the experiments; Analyzed and interpreted the data; Wrote the paper.

Sheikh Azimullah: Performed the experiments; Analyzed and interpreted the data; Contributed reagents, materials, analysis tools or data.

Khatija A. Parekh: Contributed reagents, materials, analysis tools or data.

Shreesh K. Ojha; Rami Beiram: Conceived and designed the experiments.

### Funding statement

Dr Rami Beiram was supported by 10.13039/501100006014College of Medicine and Health Sciences, United Arab Emirates University [31M411].

### Data availability statement

Data will be made available on request.

### Declaration of interest's statement

The authors declare no conflict of interest.

### Additional information

No additional information is available for this paper.
