# Supplementary material for: Effect of citronellol on oxidative stress, neuroinflammation and autophagy pathways in an in vivo model of Parkinson's disease
Source: Heliyon. 2022 Nov 3;8(11):e11434. doi: 10.1016/j.heliyon.2022.e11434 (PMC9663872; doi:10.1016/j.heliyon.2022.e11434)
Supplement: Supplementary Data.pdf [file mmc1.pdf]

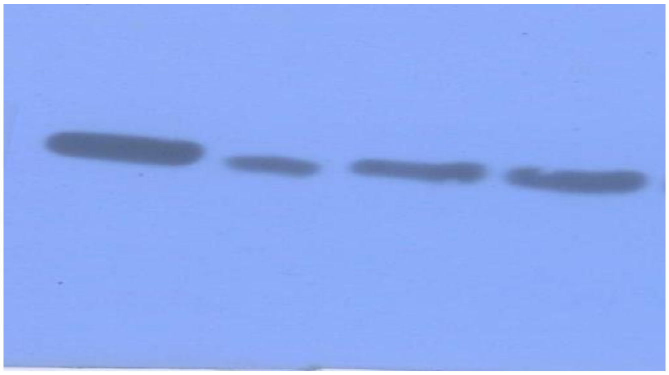

Fig 1: Full-length western blot for Nrf-2 presented in Fig.3.

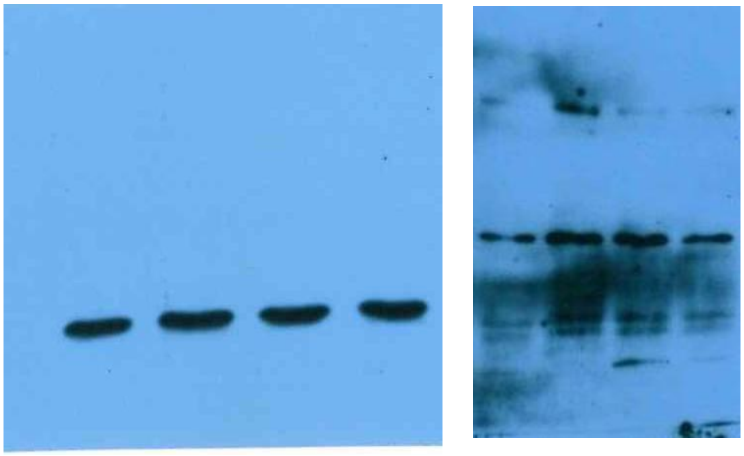

Fig 2: Full-length Western blot scans for the cropped images of Cox-2 and iNOS presented in Fig 4.

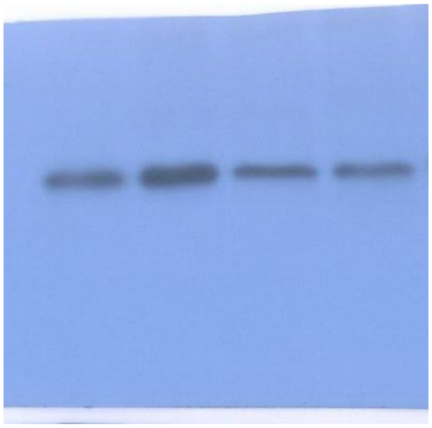

Fig 3: Full-length Western blot scan for the cropped images of α-syn presented in Fig. 7.

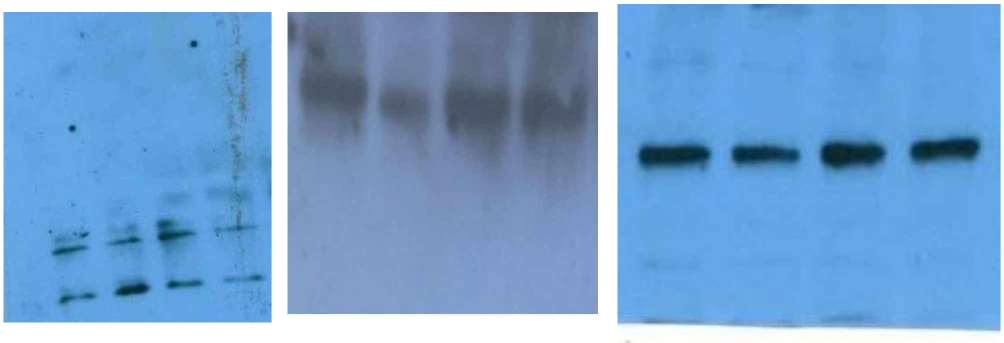

Fig 4: Full-length Western blot scans for Bax, Bcl-2 & mTOR presented in Fig. 8.

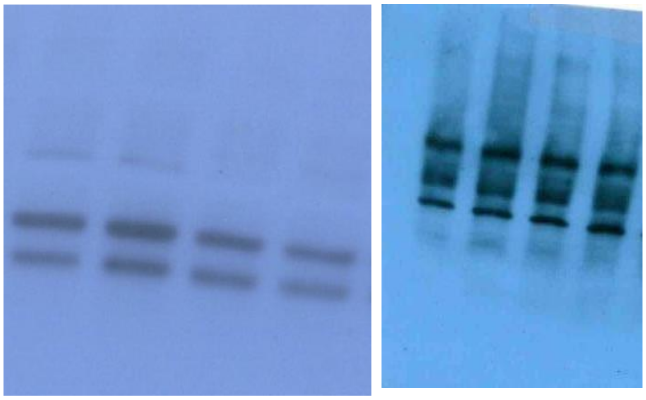

Fig 4: Full-length Western blot scans for the cropped images of LC3 and p62 presented in Fig. 9.
